# The relationship between armed conflict and reproductive, maternal, newborn and child health and nutrition status and services in northeastern Nigeria: a mixed-methods case study

**DOI:** 10.1186/s13031-020-00318-5

**Published:** 2020-11-13

**Authors:** Jennifer A. Tyndall, Khadidiatou Ndiaye, Chinwewo Weli, Eskedar Dejene, Nwanneamaka Ume, Victory Inyang, Christiana Okere, John Sandberg, Ronald J. Waldman

**Affiliations:** 1 American University of Nigeria, Yola, Nigeria; 2 Milken Institute School of Public Health, George Washington University, Washington, DC, USA; 3 Doctors of the World – USA, New York, NY USA

**Keywords:** Nigeria, Armed conflict, Humanitarian, Reproductive health, Maternal health, Child health, Nutrition

## Abstract

**Background:**

Armed conflict between the militant Islamist group Boko Haram, other insurgents, and the Nigerian military has principally affected three states of northeastern Nigeria (Borno, Adamawa, Yobe) since 2002. An intensification of the conflict in 2009 brought the situation to increased international visibility. However, full-scale humanitarian intervention did not occur until 2016. Even prior to this period of armed conflict, reproductive, maternal, neonatal, and child health indicators were extremely low in the region. The presence of local and international humanitarian actors, in the form of United Nations agencies and non-governmental organizations, working in concert with concerned federal, state, and local entities of the Government of Nigeria, were able to prioritize and devise strategies for the delivery of health services that resulted in marked improvement of health status in the subset of the population in which this could be measured. Prospects for the future remain uncertain.

**Methods:**

Interviews were conducted with more than 60 respondents from government, United Nations agencies, and national and international non-governmental organizations. Quantitative data on intervention coverage indicators from publicly available national surveys (Demographic and Health Surveys (DHS), Multiple Indicator Cluster Surveys (MICS)), National Nutrition and Health Surveys (NNHS)) were descriptively analyzed.

**Results:**

Overall, indicators of low reproductive, maternal, neonatal, and child health (RMNCH) status and intervention coverage were found in the pre-intervention period (prior to 2016) and important improvements were noted following the arrival of international humanitarian assistance, even while armed conflict and adverse conditions persisted. Security issues, workforce limitations, and inadequate financing were frequently cited obstacles.

**Conclusion:**

It is assumed that armed conflict would have a negative impact on the health status of the affected population, but pre-conflict indicators can be so depressed that this effect is difficult to measure. When this is the case, health sector intervention by the international community can often result in marked improvements in the accessible population. What might happen upon the departure of the humanitarian organizations cannot be predicted with an appreciable degree of certainty.

## Background

With a current estimated population of over 200 million people, Nigeria is the most populous country in Africa [1]. Despite having the largest economy on the continent, it lags in development and is ranked 157th on the Human Development Index [2]. There is marked inequality between the northern and southern regions of the country on all important development parameters including those pertaining to the health sector and this inequality has been growing in recent years [3]. Due to the combination of its size and relatively poor health sector performance, Nigeria is the second largest contributor to both under-five and maternal mortality in the world, with over 800,000 deaths in children under the age of five each year (30% of which occur in newborns) and nearly 20% of all global maternal deaths [4].

**Fig. 1:**
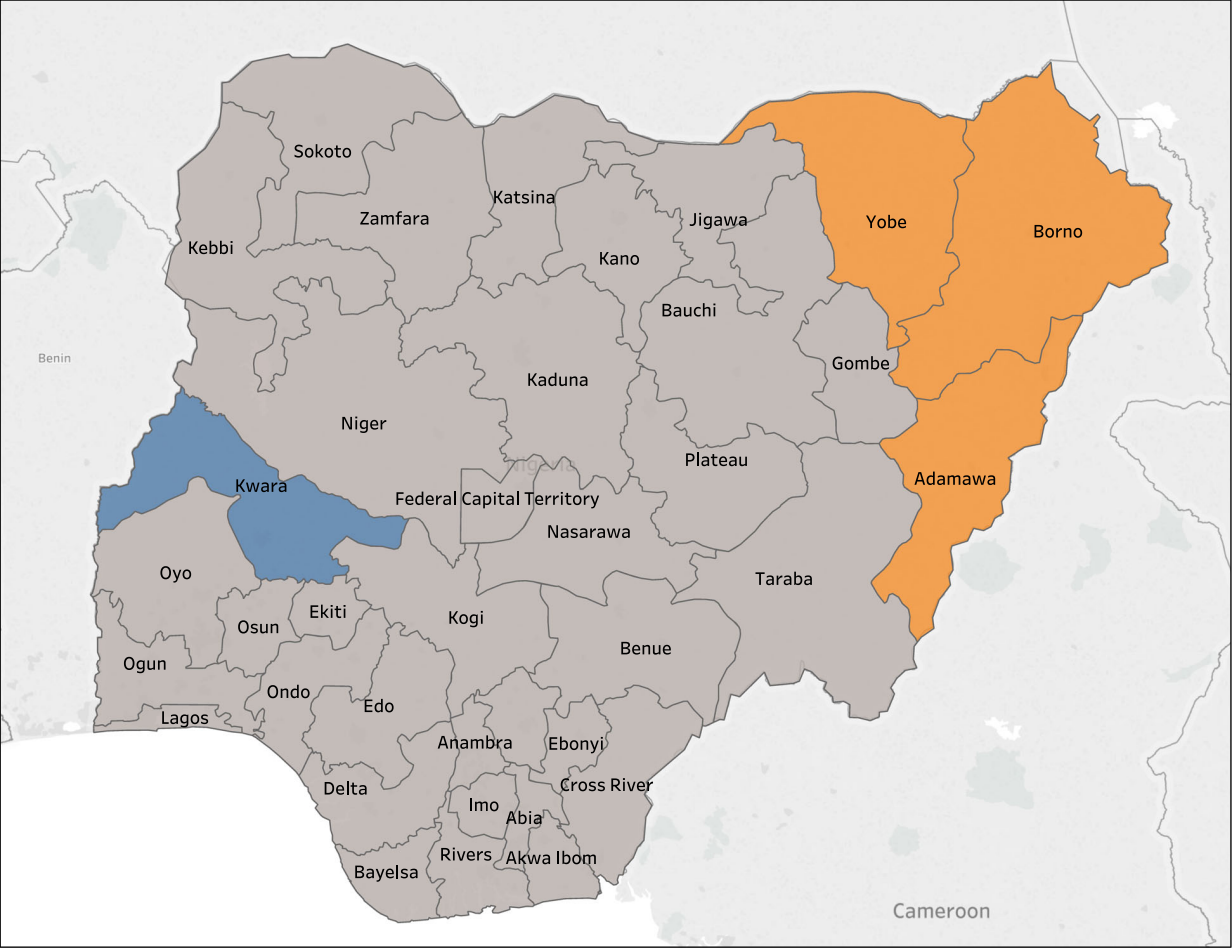
Map of Nigeria, highlighting the states included in the study. The conflict-affected states of Borno, Adamawa and Yobe are colored orange; the stable state of Kwara, used for comparative purposes, is colored purple

Social conflicts involving non-state armed groups have ravaged Nigeria for decades. These include the militancy in the Niger Delta, the Farmer-Herder clashes in the northeastern and central regions of the country and, most prominently, the Boko Haram insurgency that, while regional in scope, has been limited in Nigeria to the northeastern quadrant, primarily affecting Borno, Adamawa, and Yobe States (Fig. 1). Since 2009, the Boko Haram conflict has exacerbated pre-existing low coverage levels of health services and provoked a serious humanitarian crisis. In that year, Boko Haram, which was founded in 2002, escalated its brutal attacks in the region following the death of its leader (Table 1) [5]. Suicide bombings targeting Nigerian government facilities and personnel including the military, attacks on the United Nations (UN) installations, mass abductions such as that of the “Chibok girls”, and the extension of its activities throughout the region including in Niger, Chad, and northeastern Cameroon attracted international attention [5]. Within the northeastern quadrant of Nigeria, the lack of effective regional governance, widespread physical and food insecurity resulting in an estimated 37,500 deaths, and the physical inaccessibility and near-total absence of social services resulted in the internal displacement of more than 2.4 million Nigerians, mostly in Borno State, but also having a serious impact on the bordering states of Adamawa, Gombe, and Yobe [6].

**Table 1:** Timeline of selected Boko Haram activities

July 2009	Boko Haram uprising; 1000 soldiers killed
August 2011	Abuja Police HQ bombing; 23 killed
January 2012	New Boko Haram leader, Abu Usamtul Al-Ansari
November 2013	U.S. adds Boko Haram to its list of foreign terrorist organizations
April 2014	Boko Haram abducts 276 teenage schoolgirls in Chibok
March 2015	Boko Haram announces expansion and allegiance to ISIS
October 2016	Boko Haram hands over 21 Chibok schoolgirls to authorities
March 2018	7 UN aid workers killed, 4 abducted in Kala Balge and Rann, Borno State
April 2018	UNICEF reports Boko Haram has kidnapped > 1,000 children in northeast Nigeria
February 2019	Boko Haram attacks Shuwa, kills 3, loots and burns houses

The ongoing and rapidly growing humanitarian crisis ultimately provoked an arguably belated international response, centered in Maiduguri, the capital of Borno State, which began in earnest in 2016.

The humanitarian crisis continues to the present. As many as 7.1 million people, at least 50% of whom are children, are estimated to be in need of humanitarian assistance [7]. In Borno, a high proportion of health facilities remains inaccessible and 80% of the state is considered to be “high risk”, seriously compromising the ability of government authorities, UN agencies, and non-governmental organizations (NGOs) to deliver goods and services. Access to food, safe water, protective shelter, and health care is grossly inadequate for the population. Outbreaks of cholera, yellow fever, and meningitis have further complicated the response and, at the time of this study, the region continued to be considered endemic for polio [8].

The case study presented here is part of a set of case studies undertaken by the BRANCH Consortium (Bridging Research & Action in Conflict Settings for the Health of Women & Children) and focused on sexual, reproductive, maternal, newborn, child and adolescent health and nutrition in ten countries affected by large-scale armed conflict: Afghanistan, Colombia, Democratic Republic of Congo, Mali, Nigeria, Pakistan, Somalia, South Sudan, Syria, and Yemen.

## Methods

A generic protocol was developed in an attempt to enable cross-country comparisons among the ten BRANCH country case studies [9]. Each case study team adapted and developed a modified version of this protocol in accordance with their needs, interests, and available data. For Nigeria, the study focused primarily on maternal health, child health, and nutrition. Two study inception meetings were held, one in Abuja and one in Yola (Adamawa State), in April 2018 in order to explain the overall project to those working on both policy and program implementation at federal, state, and Local Government Area (LGA) levels of the health care system, as well as to representatives of the international community, including UN agencies, bilateral donors, and NGOs. Input received at these meetings helped to orient the research team to the realities of the context and contributed to the further modification of the research plans. A mixed-methods approach consisting of de novo collection of qualitative data and a synthesis of existing quantitative data was adopted.

### Qualitative methods

#### Sampling and recruitment

Three categories of participants were included in the Nigeria country case study based on their ability to provide informed primary qualitative data on the topics of interest. These were:

- Government (Federal Ministry of Health and/or other equivalent governing bodies, Provincial and LGA authorities);
- UN officials (representatives from OCHA, WHO, UNICEF, UNHCR, UNFPA);
- NGO upper-management (country representative/medical coordinator/logistic coordinator/security coordinator).

A purposive sampling method was used to identify the key individuals and groups involved in the delivery of health services in conflict areas of the northeast. Snowball sampling, whereby key participants were asked to refer colleagues who could speak knowledgeably on the topics of interest, was also used.

Three versions of an interview guide, one for each category of interviewee, were adapted from the generic guide developed by the BRANCH Consortium. The semi-structured guide was intended to provide information relevant to:

- The maternal, neonatal, and child health services that were offered to population groups by government and/or NGOs;
- An assessment of the decision-making processes that determined how the selection of those health services that were implemented was made: whether choices were made on the basis of available evidence of effectiveness, available funding, access to the target population, experience, convenience, or other factors.

#### Data collection

The draft interview guide was then field-tested multiple times, and the wording of questions was modified to assure their clarity for respondents. A collaborative team of faculty and students from George Washington University’s Milken Institute School of Public Health and the American University of Nigeria (Yola) conducted the interviews. All interviews were conducted in English and were recorded, with the consent of the interviewees. Interviewees were informed that no personal identifying information was being recorded, and during data entry, any information that might have led to identification of interviewees was removed. In Adamawa State, interviews were conducted in person, others were done by telephone. Each interview lasted approximately 1 h. Two authors (JT, KN) supervised all interviewees and reviewed all interview transcripts. All interviewers had completed appropriate training in the principles of ethical research and Institutional Review Board ethical approval was obtained from both institutions prior to the project being initiated.

#### Data analysis

Data analysis began during the data collection phase so that avenues of inquiry could be amended to further explore emerging issues or to resolve questions arising from the early responses. All interviews were transcribed and analysis of the verbatim transcripts was conducted in conformance with standard procedures for inductive, qualitative, and thematic analysis. NVivo qualitative software was used for data organization and analysis [10].

### Quantitative methods

Data derived from several sources representing RMNCH status and intervention coverage through different health indicators, different geographical areas, and different times were reviewed. Indicators relevant to maternal, newborn, and child health and child nutrition were purposively selected for review. These indicators were selected to represent key elements of maternal and child health that may have been the most severely impacted by the armed conflict. In addition, these indicators may be the most responsive to national and international efforts put in place to improve access to and utilization of health services in the conflict area.

Data are reported from 1) one state representing the epicenter of armed conflict (Borno); 2) one state outside of the main conflict zone whose health care system is highly affected by the emergency, to some extent by conflict, but also due to the in-migration of large numbers of internally displaced persons that place a substantial strain on its ability to provide health services (Adamawa); and 3) one state (Kwara) located in a relatively stable and conflict-unaffected part of the country that might serve as a reference for contemporary health system potential in Nigeria. State indicators are compared to national averages when appropriate.

The time series covered here imperfectly captures the period immediately prior to the onset of intensified armed conflict (pre-2009), a period of escalating conflict prior to the onset of major assistance programs reinforced by the international community (2000–2014), and the most recent period of intensified international assistance (2015–2018). The data points in these series are population estimates from separate surveys conducted over the period in 2008, 2011, 2013, and 2014–17.

Estimates from 2008 and 2013 are derived from the Nigeria Demographic and Health Surveys (NDHS) [11, 12] and those for 2011 and 2016 are derived from the Multiple Indicator Cluster Surveys (MICS) [13, 14]. Estimates from 2014, 2015, and 2018 are derived from the National Nutrition and Health Surveys (NNHS) of those years [15, 16] as well as from the 2018 Demographic and Health Survey [17].

The NDHS, MICs, and NNHS are nationally representative household surveys that use identical methodologies for collecting information on RMNCH and other outcomes. The DHS program, funded by the United States Agency for International Development and implemented by Macro International implements standardized surveys throughout lower and middle-income countries, as does the MICS, funded and implemented by UNICEF. Both programs work closely to harmonize methodologies and indicators used. The NNHS is conducted by the Nigerian government with technical support from UNICEF, and was designed specifically to allow for comparison to DHS and MICS results [14, 18–20].

We also reviewed additional data from relevant websites and indexed publications. No primary quantitative data were collected during the course of this study.

## Results

### Qualitative

#### Participants

Sixty-one (61) interviews were completed with participants drawn from the government, United Nations agencies, and both international and local non-governmental organizations (Table 2).

**Table 2:** Number of interview participants by organization

Organization	Participants
Government officials	13
United Nations agencies representatives	9
NGO representatives	39
**Total**	**61**

When responses from the interviewees were analyzed a number of common themes became apparent. The most prominent of these were the interplay between the conflict and concerns regarding both health worker and beneficiary security, issues concerning the health workforce, the ability to pay for program operations, and the process by which decisions were made regarding which interventions to implement and how to implement them.

#### Security

Security concerns secondary to ongoing, albeit sporadic, armed conflict are one of the most prominent issues surrounding the provision of health services. Complications emerging from insecurity that were most frequently mentioned included the difficulty of establishing a target population due to constant population movement, gaining access to distant populations in an insecure setting, and the perception of more frequent outbreaks of communicable diseases. One Government representative said:

“It has affected me because there is no way we can go to some areas due to the security challenges. Some of the people have left the place. So this affects the delivery of my services.”

Participants tended to focus on different aspects of security and the way they affected service delivery. The need to be accompanied by guards, the difficulty of obtaining accurate information, and the psychological toll of learning of attacks on service providers were frequently mentioned. A UN official talked about this:

“… first we must be given clearance by the DSS [security department]. Written clearance. If they don’t give, we don’t go. That apart, we trust as much as possible to maintain a low profile. Personally, in areas that are overcrowded, we don’t go there. And then we’ll hear there is conflict in a particular area, we try to avoid it. By so doing, I’ve survived since I’ve been here …”

However, a few participants did not see the security situation as a general deterrent. Still, even in their confidence, considerable hesitancy is present. As a government official put it:

“...if you’re outside of here, and hear about it, it’s like it’s a war zone, bombs are falling on top of everybody. But if you’re here, you see a little bit different. It’s not that there are no challenges, there are, but it’s still bearable.

Even when organizations did feel able to work relatively safely, this was usually only within limited geographical areas. These changed frequently, and what was a safe zone 1 week might be a “no-go” area the next.

While the circumstances might be difficult for the larger organizations, such as UN agencies, or even for international NGOs, for less experienced local NGOs, ones that had formed to meet the needs of the current crisis, the situation was that much more difficult:

“when we get to Maiduguri of course we ask questions and we meet with other organizations. They tell us what areas they think we can access. They know we are from a small NGO that is just starting. We do not have, let me put it this way, the bulletproof jeeps … so they put all of that into considerations and give us a specific location to focus on. Based on this advice, we restrict ourselves to the safe areas.”

The stress of working in an insecure environment took a toll not only on organizations and their humanitarian operations, but on the individual humanitarian workers as well:

“The crisis made me lose touch with myself; it made me focus mainly on other people. I had short sleep at night, my social life diminished. I avoided going to church because security reports made me think of the church as a target.” (an NGO worker).

Security issues were closely linked to the inability of organizations to perform to their highest ability:

“The security situation is very hard because, in the last two years, the organization has not done well because of the fear of unknown places and safety of staff members making our work to drop. The level of acceptability has dropped because of the insurgency as most people are afraid of strangers.”

“In some instances, some LGAs were not reached with interventions due to the security challenges.”

Most (> 55%) personnel from UN agencies stated that staff from their respective agencies had experienced episodes of violence during the humanitarian interventions. It is important to note that while the larger international agencies could offer their personnel liberal rest and recreation policies to help ease the mental and emotional stress, local health workers and NGOs do not have the same benefits or, in many instances, the same working conditions.

#### Health care workforce

Closely related to the security issue, especially to the last few paragraphs above, is that of a depleted workforce. This was seen as a major limitation on the ability to deliver services. Factors such as safety, especially for female service providers in the prevailing culture, professional qualifications, and concerns about the level of skill of some health workers all contributed to a lack of ability to recruit adequate staff. An NGO staff person said:

“The staff members are not readily available as most newly recruited staff members refused to accept their appointments because of the volatile nature of the places they were posted to.”

One UN participant said:

“The population we work with is quite sensitive to the extent that men … do not mix with females in these locations [in northeast Nigeria]. So, whenever we’re implementing our programs … they all have to be served separately … unlike other locations [in Nigeria] where you find this being a very normal practice.”

Participants also talked about the number of local staff available. Some government officials added that they would prefer health workers who were familiar with the area and with the people, but in some areas many of the health workers had left because of recurring insecurity. One said:

“We want healthcare workers from specific geographical locations, but they are not available. Mobilizing people from other regions is often difficult because they don’t stay. We have a challenge of getting indigenous health workers.”

NGOs also cited problems with health staff retention.

“Availability of competent staff is the problem. All NGOs have a problem of turnover – train staff and they find a better job and leave, especially from the local NGOs.”

#### Funding

Insufficient financial resources led partners to compromise certain planned interventions and forced what was considered to be an excessively limited “triage” or “prioritization” of the most urgent needs. The availability of funds, or lack thereof, was frequently cited by study participants as one of the most important factors (in the eyes of some, more so than security or workforce limitations) that impeded needs-based decision-making. One local NGO worker put the relationship between funding and programming succinctly:

“When there is funding, we work; when there isn’t, there is nothing we can do.”

Another said:

“… because funding is never enough, that usually influences our decision in the area of how we have to prioritize the most urgent and critical and lifesaving interventions. Others are interventions that … they can wait. Yeah, they can wait and those that can wait, wait.”

The funding situation for the northeastern Nigeria crisis is difficult to assess and to track over time. While it has been called “one of the world’s best funded” humanitarian responses in 2017, this is a relative statement, as ‘only’ 71% of the USD 1.05 billion funding requirements of the annual Humanitarian Response Plan had been received [21]. Because the details of the amount requested and actual expenditures are difficult to ascertain, and because the number of beneficiaries fluctuates widely on the basis of accessibility, actual cash flow to the field of operations is often constrained. In fact, food assistance decreased for over 2 million people in the area by the end of 2017 and the same paragraph from the OCHA report cited admits that “a critical gap … hampered the humanitarian agencies’ ability to deliver comprehensive livelihood support to affected people …” [21, 22].

#### Decision-making processes

Participants focused on the means by which decisions were made regarding which interventions in the RMNCH arena should be, and could be, implemented. They pointed to:

- National factors

Respondents generally agreed that the Government of Nigeria, both at federal and state levels, plays a central role in the humanitarian intervention, both through the establishment of policy and, importantly, in influencing the relationship with other stakeholders. Still, it must be recognized, this is being done while the Government is still a party to the armed conflict. There was widespread agreement that Government laws and regulations, especially in areas of active armed conflict, must be respected.

Similarly, there was agreement that the decision-making process was multifactorial and not entirely evidence-based. Politics plays an important part. Many players assert, or try to assert, authority in the conflict-affected areas of the northeast (federal, state, and LGA officials, in addition to international groups including a host of UN agencies, donors, and NGOs). Interviewees expressed the sentiment that they needed to appear neutral and to not oppose the Government in any way in order to be viewed as a cooperative partner. Some saw this as requiring compromise. An NGO representative said:

“A challenge you are going to face working in the northeast is the “body language” of the Government. The Government is a partner, but it is also a very difficult partner. While you have good intentions, [the government] wants to align their actions with their political ambitions and their political agenda.”

Another participant felt that as much as it was important to work with the Government, this could have negative consequences for the health programs in areas where there was widespread mistrust of Government authority:

“So, still you think toward their political agenda and, if you are not careful with that, the people you want to help will see you as part of the Government. So that becomes a problem. So, you are no longer accepted in communities because people see your work as politicized. And they begin to lose trust in what you are doing. So, managing that government relationship is critical.”

b) International factors

Inter-institutional and inter-sectoral coordination are fundamental to health sector interventions. In most cases, non-governmental organizations and the UN agencies work very closely at the national, provincial, and local levels to develop health sector strategies and to optimize health intervention service delivery. The Health Cluster has consistently received high marks from all participants who were asked about it and all agreed that coordination and cooperation between agencies has been useful and effective.

That said, NGO decision-making and prioritization were found to correlate highly with the mandate and focus of the organization. Organizations tended to stay in their “lane” – what was deemed to be of highest priority to them was what they did as part of their mandate, not necessarily what was most needed in a given place at a given time.

### Quantitative

RMNCH status and intervention coverage indicators, derived from the existing quantitative data sources mentioned above, were examined by state before the Boko Haram insurgency, during the period of conflict intensification, and after the start of the international humanitarian relief effort. To further highlight the evolution of health service delivery and health status of the population over time, trends in a subset of selected indicators are presented below, by state and year, and compared to the national average.

**Fig.2:**
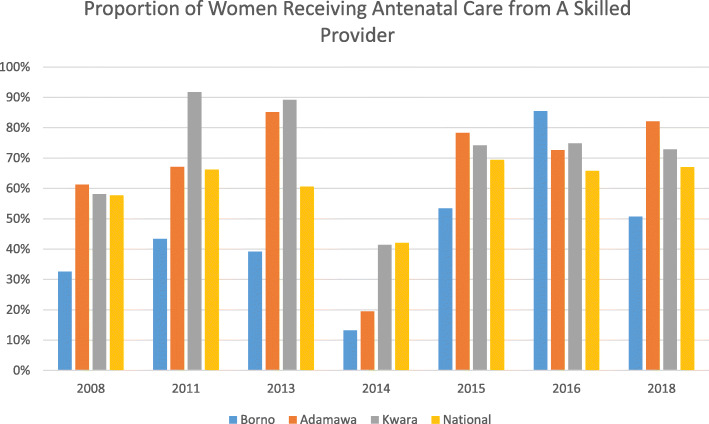
Proportion of Women Receiving Antenatal Care from A Skilled Provider, by Selected State and by Year, Compared to National Average

With respect to access of pregnant women to skilled antenatal care, Fig. 2 shows the estimated proportion of pregnant women who had at least one antenatal care visit.

Here we see a relatively high level of coverage (> 50%) over time in Adamawa and Kwara, interrupted by a steep decline reported by the 2014 NNHS. Though this decline may be real, estimates decline dramatically for all states, and nationally, suggesting that this may be an artefact associated with the survey. Antenatal care coverage in Borno State, in contrast, which started from a much lower level than in either of the other two states under study, appears to have increased dramatically in 2015 and 2016, approaching parity with the other states in 2015 and surpassing both them and the national average in 2016, the year that the international humanitarian intervention began in force. The steep decline of this indicator in Borno in 2018, while it remains relatively stable in the other states is difficult to explain.

**Fig. 3:**
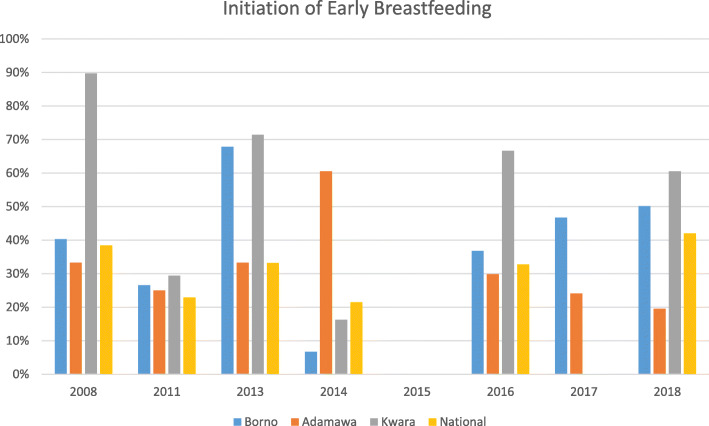
Initiation of Early Breastfeeding, By Selected State and Year, Compared to National Average

In regard to initiation of early breastfeeding (within 1 h of live birth), data limitations reduce the level of confidence one can have in making inferences concerning trends across this series of data points (Fig. 3). Estimates were not available for this indicator for 2015 and, as discussed above, the estimate for 2014 appears to be anomalous relative to other years.

Taking these factors into account, the prevalence of early breastfeeding initiation appears to be highest in Kwara State, the control state for this analysis. Prevalence in Borno and Adamawa States is similar, except for the 2013 DHS, where the data from Borno appear to indicate an unexpectedly high level of achievement, and the indicator is again quite low in the 2014 data, except for Adamawa State, where it is unexpectedly high.

**Fig. 4:**
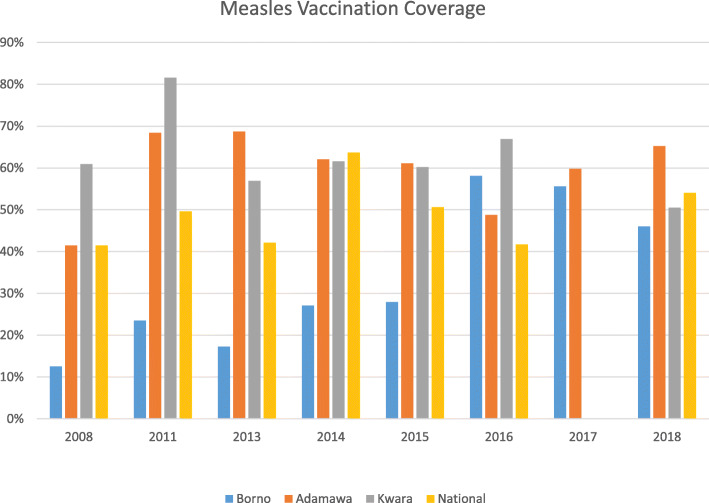
Measles Vaccination Coverage, by State and by Year, compared to National Average

An important indicator of child health service delivery, measles vaccination coverage in Borno State is seen to be abnormally low (12.5%) in 2008, prior to the intensification of the Boko Haram insurgency, and remains at a relatively low level, increasing slowly through the 2015 survey. There is a major improvement in 2016, to 58.1% in 2016, when measles vaccination coverage in both Borno and Adamawa States exceeded the national average for this indicator (Fig. 4). In Adamawa State, coverage was estimated to be relatively higher in than the national average at the beginning of the time series and remained relatively stable, with a slight decline from 2011 until 2017. The 2018 DHS shows measles vaccination coverage in the northeastern states to be relatively similar to the control state and to the national average.

**Fig. 5:**
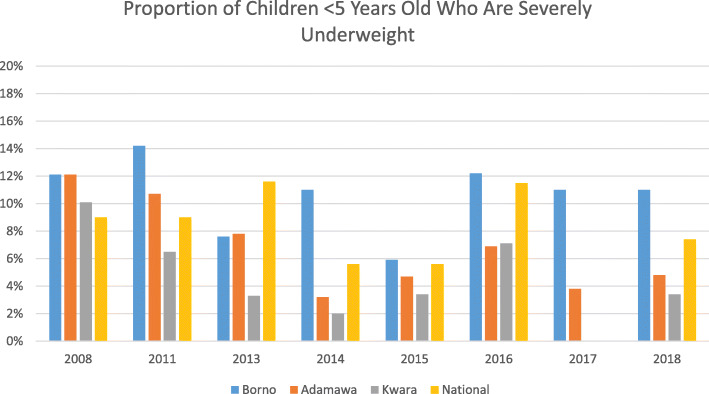
Proportion of children < 5 years old who are severely underweight, by State and Year, Compared to National Average

Finally, the estimated proportion of children less than 5 years of age identified as being severely undernourished (equal or greater than 3 standard deviations below the reference weight for age) revealed a disparity between Borno State and the others examined, as well as an important difference between Borno and the national average (Fig. 5). Excluding the estimate of the 2015 SMART survey, the prevalence of severe undernutrition in Borno does not appear to have declined to same extent as in the other states.

However, the results of these small-scale surveys are somewhat contradicted by surveys conducted in Borno and Yobe States by the Nigerian National Bureau of Statistics, in coordination with the Federal Ministry of Health, United Nations Children’s Fund (UNICEF), and the US Centers for Disease Control and Prevention (CDC) in October–November 2016 and February–March 2017. Despite showing improvements in the prevalence of global acute malnutrition and in measles vaccination in most areas surveyed, both crude and under-5 year mortality increased from the first to the second round of the surveys and remained above the emergency threshold in parts of both states [23]. The authors of this report state that while interventions aimed at reducing maternal and child undernutrition and mortality had been prioritized, their “survey results indicate that substantial gaps in use of these important interventions remain.” They further point out that, given their surveys were limited by inaccessibility to a substantial proportion of the population, the situation could have been even worse at the time.

In general, a pattern that is repeated across all indicators available from the national surveys conducted is that the northeastern states, and Borno State in particular, begin the time period examined with poor, sometimes extremely poor, indicators of health status and health service delivery in the RMNCH arena. Over the course of time, and through 2018, where data is available, one generally sees a substantial improvement in the indicators in Borno State which approaches, or in some cases surpasses, the national average. The inferences that can be drawn from this observation will be discussed in the next section.

## Discussion

If the purpose of this case study were to measure the impact of armed conflict on selected RMNCH indicators in northeastern Nigeria, one would have a difficult time. At the time of the intensification of Boko Haram activities in 2009, many of the indicators measured by a national survey (DHS) were already seriously depressed. Nigeria, despite having the second largest economy in Africa [20], has, for decades, been cited as being among the countries with the most unequal distribution of wealth, having a Gini coefficient ranking of 152nd (World Bank, 2013; “World Development Indicators 2013” Washington, DC). There are clear spatial elements to the maldistribution of wealth, with the northeastern states being among the most deprived, dating from long before the Boko Haram uprising.

This circumstance suggests that the armed conflict in Nigeria might share characteristics with some of the other countries included in this set of case studies such as Somalia, South Sudan, Afghanistan, and perhaps Yemen and the Democratic Republic of Congo, but is distinguished from all but the last in that the conflict, and the domestic and international response to it, is confined to one geographic region of the country, rather than being national in scope. Nigeria can also be distinguished from conflict-affected countries like Syria or the former Yugoslavia during the 1990s, in that its conflict is not associated with a steep negative change in health indicators from previously acceptable or near-acceptable levels. Instead, a health sector response of any degree of effectiveness could only have resulted in an improved health and nutritional status of the population, as is clearly seen. Still, ongoing conflict, with associated security issues, limited access to the affected population, massive internal displacement, and an inadequate health workforce, as well as less-than-optimal funding, all contribute to vitiating the beneficial impact of the response.

Although intensification of the Boko Haram insurgency had begun in 2009, more than 3000 people were estimated to have died by the time President Goodluck Jonathan first declared a state of emergency in Borno, Adamawa, and Yobe States in response to the conflict in May 2013. (Earlier measures had applied only to a few local government areas.) The seriousness of the situation was similarly relatively ignored by the international community, United Nations agencies and non-governmental organizations alike. A belated, and somewhat timid, response to the situation was not mounted in earnest until after highly-publicized events such as the kidnapping of the “Chibok girls” in April 2014. Still, it was only in 2015, after massive forced migration from Nigeria to Cameroon, Niger, and Chad and internal displacement to the Maiduguri area (the capital of Borno State) revealed the extent of the humanitarian crisis that serious attention was attracted. The reasons for this late response are complex and have their roots in longstanding political, social, and economic relations both within Nigeria and in its relations with the external world, but the extremely difficult security situation definitely played an important role. From late 2015 through the first quarter of 2016 no humanitarian assistance was delivered beyond Maiduguri due to a combination of unpredictable Boko Haram attacks and the counter-terrorism activities of the Nigerian military forces [24].

The health sector response to the crisis appears to have been reasonably well coordinated, both in Abuja, the capital, and with a World Health Organization (WHO) coordination team working in Maiduguri in close association with the Borno State Government, the State Primary Health Care and Development Agency, the National Emergency Management Agency, and UN and INGO partners. The first bulletin of the Borno State Health Sector response was issued in September 2016 and listed 17 health sector partners who had provided over 600,000 medical consultations. Remarkably, more than half (334/632, 52.8%) of existing health facilities in the state were heavily damaged, looted, or closed and a similar proportion of population settlements were also inaccessible. It is clear, though, that resources were grossly insufficient to provide a satisfactory level of services even to the approximately 1.5 million internally displaced people who could be reached.

While there is no account of how health and nutrition interventions were selected and prioritized by the sector coordinating group, a partial list of services being provided by different agencies is available.

Although it states that “direct lifesaving assistance targeting the most vulnerable in the most affected areas remains the focus of the humanitarian response in Borno State …” , an intervention of very high priority to the responders appears to have been the implementation of a mass polio immunization campaign. Nigeria, along with Pakistan and Afghanistan, was, at the time, one of the three remaining countries in the world considered to be endemic for wild poliovirus. In August and September 2016, four cases of type 1 wild poliovirus were reported from Borno State and it is clearly understandable that health authorities would respond, although other health issues may have been of greater priority to the affected population. The effectiveness of even these massive vaccination campaigns was limited by the level of inaccessibility to the population shown on the map above.

Other concerns mentioned at this early time of the response include clinic-based and mobile primary health care services (it is not specified whether these were primarily preventive or curative in nature), sexual and reproductive health services, and psychosocial and mental health services.

Reacting to the situation as it was found to exist in the early stages of the international response, a Health Sector Response Strategy (HSRS) for 2017–2018 was developed [25]. This strategy was intended to be implemented throughout the northeast region, to the benefit of the 6.9 million affected people in Borno, Adamawa, and Yobe States. The strategy is consistent with the National Health Sector Response to Humanitarian Crisis Plan for the Northeast of the Federal Ministry of Health as well as with the state-specific operational plans.

The single goal of the HSRS was to “reduce mortality and morbidity through provision of lifesaving essential health services”, and four sub-goals are specified:

- ■ To reduce crude mortality rate to below 1 death/10,000 population/day
- ■ To reduce under-5 mortality rate to below 2 deaths/10,000 under-fives/day
- ■ To reduce under-5 global acute malnutrition to below 10% of children under 5 years
- ■ To interrupt wild poliovirus type 1 (WPV1) transmission

In addition to the specified goals, three over-riding health sector objectives are detailed: 1) to provide access to lifesaving and life-sustaining humanitarian health assistance to affected internally displaced persons (IDP) and host community populations; 2) to establish, expand and strengthen communicable disease surveillance, outbreak prevention, control and response; and 3) to strengthen health sector coordination and health information systems to improve the live-saving response for people in need.

To achieve these objectives, the coordination group agreed upon a minimum package of health interventions, similar to the Basic Package of Health Services originally pioneered successfully in Afghanistan and subsequently replicated in a number of other humanitarian settings [26]. The basic package of services adopted by the Nigeria coordination team was heavily skewed towards RMNCH interventions and consisted of the following:

- childhood vaccinations
- integrated management of childhood illnesses (IMCI), with a particular focus on malaria, pneumonia, malnutrition and diarrheal diseases
- maternal and child and neonatal health (MCNH), including both emergency and basic obstetric and newborn care (EmONC and BEmONC)
- reproductive health including HIV services and GBV programming
- management of common conditions including non-communicable diseases
- delivery of mental health and psychosocial services
- strengthening referral systems

The strategy aspired to provide all of these services through a network of Primary Care Centers, Primary Care Clinics, and Health Posts within each LGA, with one secondary facility per LGA to which cases could be referred from the more peripheral levels. Clear criteria for prioritizing the support to facilities are listed and that support is conditioned upon an acceptable security environment, available manpower, and adequate financing, acknowledging the need for physical reconstruction of damaged and destroyed sites. The design of the proposed system is logical and consistent with best practices. As seen above from both the qualitative and quantitative data available, implementation of this strategy was greatly hampered by the harsh realities of the humanitarian context.

In addition to the strategy, aspirational indicators of achievement are also specified in the HSRS. Relevant to RMNCH, these include:

- % of children <5 years old receiving PHC services: 80%
- % of deliveries attended by skilled health workers: 40% (this was the national average at the time the HSRS was developed)
- % of children < 5 years old with severe acute malnutrition receiving appropriate in-patient treatment: 75%
- % of children vaccinated against polio (number of doses unspecified): 95%
- % of children < 5 years old vaccinated against measles: 95%

We have gone into considerable depth in describing the HSRS in this narrative in order to demonstrate that coordination of the relief effort appears, at least to the outside observer, to have been strong and effective. Nigerian federal and state authorities, UN agencies (and specifically the World Health Organization and Unicef), and international and national non-governmental organizations were able to develop a consistent “game plan” for what services would be developed and delivered. The level of detail of the selected priorities is perhaps more general than what one might optimally desire, e.g., what specific vaccines would be/could be offered, what specific proven-effective MNCH and reproductive health services would be generally available throughout the region, but given the level of baseline indicators and the difficult access to the affected population, the content of the minimal package of interventions seems quite reasonable and perhaps a bit over-ambitious.

### Surveillance

Being able to collect useful, representative, and timely data on a regular and consistent basis poses a problem in all humanitarian settings. In order to help improve the situation in northeastern Nigeria health sector authorities adapted and implemented, in 2016, the World Health Organization’s Early Warning Alert and Response System [27]. While the stated objective of the EWARS is to improve outbreak detection during an emergency, the system is equally useful for the detection and enumeration of endemic diseases, and, by becoming part of the Nigeria IDRS (Integrated Disease Reporting System), has proven capable of improving upon the previously existing, but largely ineffectual, passive reporting system of the Ministry of Health. Relevant to the RMNCH focus of this report at least in part, the EWARS in Borno State was designed to report on the following, among other conditions: malaria, acute respiratory infection, acute watery diarrhea, measles, and severe acute malnutrition. Since its advent, monthly Health Sector Bulletins have been produced by the coordination group and results of the EWARs are published in each one. At first, the EWARS covered 56 health facilities in 16 camps for internally displaced persons (IDP) in 5 LGAs and, as of this writing, it had expanded to cover 278 reporting sites in 32 IDP camps in 23 LGAs, covering approximately 1.5 million people [28]. The system has been successful in reporting annual outbreaks of measles in Borno State, as well as in reporting regularly on the number of cases of presumed malaria and acute respiratory infections.

Another important element of the emergency surveillance system is the Health Resources Availability Monitoring System (HeRAMS) [29]. Established by WHO and the Global Health Cluster, HeRAMS provides information regarding potential access of a population to facility-based health services. Information is collected from four principal domains: 1) the number, location, and functionality of health facilities; 2) the human resources and equipment required for service delivery; 3) the availability of services, including sexual and reproductive health, maternal and newborn health, child health, and communicable disease control, among others; and 4) an analysis of the reasons for sub-standard performance, including physical constraints, insufficient staff, lack of equipment, inadequate financing, among other factors.

HeRAMS was established in northeastern Nigeria in 2016, with information collected by means of cross-sectional surveys in the three most affected states. For Borno State, 35% of 743 health facilities were found to have been completely destroyed and an additional 30% were partially damaged. The situation in Adamawa and Yobe States was found to be somewhat better, with 12% of facilities completely destroyed in the former, and 10% in the latter. Given this level of destruction due to the ongoing conflict, it is understandable that health service delivery from fixed facilities was severely compromised, consistent with the finding of depressed health indicators, especially in the RMNCH arena. The January 2019 Health Sector Bulletin cited presents data from the September/October Borno State HeRAMS reporting that 375 (50%) of 755 health facilities were still non-functional with 39% of those “fully damaged”, 27% “partially damaged” and only 34% “not damaged”. Clearly the situation regarding health facilities has not improved although the RMNCH status of the population appears to have made considerable progress, perhaps because much of the population has become more accessible due to displacement towards more secure population centers. The HeRAMS continues to serve a useful function.

As mentioned above, the selection of priority intervention areas appears to have been done in a coordinated and systematic way. In humanitarian emergencies, however, it is not only the interventions that have to be carefully considered and selected on the basis of priority needs but their means of delivery as well. Where routine services are not readily available to a vulnerable population, as might be the case when health facilities are non-functional (as evidence by the HeRAMS data), alternatives must be found. Two examples from Nigeria might be considered.

The first example of an alternative means of health service delivery is one of procedure. Considering the results of the HeRAMS, which showed widespread destruction of fixed health facilities with a resulting major loss of access, mobile “hard-to-reach” (HTR) teams were developed throughout northern Nigeria in 2015, initiated by the polio eradication program [30]. For the humanitarian response, an HTR-MNCH project was initiated, with trained health care workers traveling to accessible but underserved areas for short periods of time. Services such as childhood vaccinations, malaria detection and treatment, vitamin A supplementation and deworming, screening for nutritional status and ante- and post-natal care are offered in temporary, makeshift clinics on a periodic basis.

A second example, one of a modified technical approach, addresses the burden of malaria. A strategy of seasonal malaria chemoprevention (SMC), introduced by the World Health Organization in 2012, is considered to be potentially effective given the highly seasonal transmission pattern in the Northeast. Mass drug administration of a combination of amodiaquine and pyrimethamine/sulfadoxine to children aged 3–59 months in 3 monthly doses during the high transmission season could have a greatly beneficial impact, especially if high levels of coverage are achieved [31]. Implementation of SMC began in Borno State in 2017 and a positive impact was demonstrated. In 2018 implementation was planned throughout the northeast, aiming to reach over 800,000 children in Borno State alone, using hundreds of trained volunteers [32]. These two examples of innovative means of expanding health service delivery to a maximum of conflict-affected people, even where the traditional health-facility-based strategy in inoperable, can serve as models for other humanitarian settings.

Finally, a number of serious outbreaks of communicable diseases have disrupted, to a degree, the ability of the humanitarian health community to provide essential primary health care services as initially intended. In early 2017, for example, preparations were made for the eventuality of an epidemic of meningococcal meningitis, one that had already been responsible for more than 5000 cases across the northern states of Nigeria. The Health Cluster assisted the State Ministries of Health and Primary Health Care Agencies of Borno, Adamawa, and Yobe States in the development of Epidemic Preparedness and Response plans that call for the training of Rapid Response Teams, among other interventions [33]. In addition, from the start of 2017, all three conflict-affected states in the northeast were seriously impacted by an outbreak of cholera which was spreading across northern Nigeria. In Borno State, a coalition of national and international agencies and organizations, coordinated by the Emergency Operations Center of the State Ministry of Health, responded. In 5 LGAs where the outbreak was particularly severe, over 5000 cases of cholera were recorded, but the overall case-fatality rate was 0.87% [34]. This result was better than what was achieved in other, non-conflict-affected states in neighboring areas, suggesting once again that the presence of the humanitarian organizations in the northeast had a positive impact on population health.

The polio outbreak of 2016 and the mass vaccination campaigns in response to it and to the threat of measles are mentioned above. As of this writing, in late 2019, an important measles outbreak, with cases numbering in the thousands, is occurring in the region and throughout the country [35].

## Conclusion

This case study of the humanitarian health situation in Northeastern Nigeria is illuminating in a number of important ways. For one thing, it highlights the fact that many populations living in conflict-affected areas were suffering from low levels of health and other social services, resulting in inferior health status, from long before the onset of armed conflict. Many humanitarian crises occur in areas that have been neglected by national and regional governments, and northeastern Nigeria, as the data can attest, is among them. When this is the case, it is evident that international humanitarian assistance can only result in an improvement, sometimes a radical improvement, in health status, at least for those who can be reached. In Nigeria, and especially in Borno State, the humanitarian response resulted, for example, in a sharp increase in childhood vaccination status from the exceedingly low levels that existed even prior to the intensification of armed conflict and Boko Haram activities to levels that rivalled and, in some instances, surpassed the national averages. This is obviously a good thing, and a testament to the dedication and effectiveness of both local and international humanitarian organizations, as well as to Government authorities who, during this time, intervened in the health and other sectors.

Nevertheless, the success of the current humanitarian intervention by no means guarantees that, should the intensity of the armed conflict recede, or should a lasting peace agreement be reached, the contemporary gains will hold into the future. Indeed, a return to the *status quo* ante bellum*,* to a situation characterized by unacceptable health services and health status, would be devastating. Still, for now, a well-coordinated humanitarian response, combined with innovative approaches to RMNCH service delivery, has yielded positive results even in the face of serious political, manpower and financial constraints. For the results to become long-lasting, an effective transitional strategy, characterized by a major commitment from the federal, state and local levels of the Nigerian Government, as well as by a durable presence of external funding and technical assistance will be required [36].

### Data limitations

As has been stated several times in this report, the impact of security issues on the ability to collect accurate data in a way that is representative of the entire target population is major. Even the “representative” national surveys could not always ensure representativeness. For example, the 2013 DHS was unable to collect data from eight of the clusters that were selected for its sample [12]. As is pointed out, ongoing insecurity leads to constant population migration, partial or total destruction of health facilities and, therefore, of service delivery, and impedes the ability of mobile teams to reach people in need. For these reasons, the quantitative data relied upon here may not reflect a totally accurate account of the health status of the population. Also for security reasons, the study team was unable to travel to Borno State. Interviews conducted with subjects in Borno were all done by telephone. In contrast, interviews in Adamawa State, where the America University of Nigeria is located, could frequently be done in-person. It is unlikely that this difference affected the integrity of the data.

## Data Availability

Data available upon reasonable request.

## Abbreviations

BRANCH: Bridging Research and Action in Conflict Settings for the Health of Women and Children
NGO: Non-government organization
UN: United Nations
MICS: Multiple Indicator Cluster Survey
DHS: Demographic and Health Survey
LGA: Local Government Area
NDHS: Nigeria Demographic and Health Survey
OCHA: United Nations Office for the Coordination of Humanitarian Affairs
WHO: World Health Organization
UNICEF: United Nations Children’s Fund
UNHCR: United National United Nations High Commissioner for Refugees
UNFPA: United Nations Population Fund
RMNCH: Reproductive, Maternal, Newborn Child Health
NNHS: National Nutrition and Health Survey
SMC: Seasonal malaria chemoprevention
HeRAMS: Health Resources Availability Monitoring System
IDRS: Integrated Disease Reporting System
HTR: Hard-to-reach
MSF: Médecins Sans Frontières
EmONC: Emergency Obstetric Newborn Care
BEmONC: Basic Obstetric and Newborn Care
IMCI: Integrated management of childhood illnesses
PHC: Primary Health Care
GBV: Gender-Based Violence
CMR: Crude mortality rate
IDP: Internally displaced people
HSRS: Health Sector Response Strategy
U5MR: Under-five mortality rate
WPV1: Wild poliovirus type 1

## References

[1] World Population Review (2020). Nigeria Population.

[2] United Nations Development Programme (2020). Human development reports: Nigeria.

[3] World Bank (2019). The World Bank in Nigeria 2020.

[4] World Health Organization (2019). Maternal health in Nigeria: generating information for action.

[5] Matfess H (2017). Boko Haram: history and context Oxford research encyclopedias.

[6] Council on foreign relations (2020). Global conflict tracker.

[7] United Nations Office for the Coordination of Humanitarian Affiars (2019). Nigeria: 2019 humanitarian needs overview.

[8] World Health Organization (2020). Emergencies preparedness, response.

[9] Ataullahjan A, Gaffey MF, Sami S, Singh NS, Tappis H, Black RE, et al. Investigating the delivery of health and nutrition interventions for women and children in conflict settings: a collection of case studies from the BRANCH Consortium. Conflict Health. 2020;14(1). 10.1186/s13031-020-00276-y. 10.1186/s13031-020-00276-y PMC7254714 32514294

[10] QSR International (2020). NVivo.

[11] National Population Commission (NPC) [Nigeria] and ICF Macro. Nigeria Demographic and Health Survey 2008. Abuja: National Population Commission and ICF Macro; 2009.

[12] National Population Commission, ICF International, ICF Macro (2014). Nigeria demographic and health Survey 2013. National Population Commission.

[13] National Bureau of Statistics, United Nations Children's Fund, United Nations Population Fund (2013). Nigeria multiple indicator cluster survey (MICS) - 2011: Monitoring the situation of children and women.

[14] National Bureau of Statistics (2018). Multiple Indicator Cluster Survey (MICS5) 2016–17, fifth round.

[15] National Population Commission (NPC) [Nigeria], ICF (2019). Nigeria demographic and health survey 2018.

[16] National Bureau of Statistics – Nigeria (2015). National Nutrition and Health Survey, 2015.

[17] United Nations Children's Fund (2018). National nutrition and health survey (nnhs) 2018.

[18] Corsi DJ, Neuman M, Finlay JE, Subramanian SV (2012). Demographic and health surveys: a profile. Int J Epidemiol.

[19] Hancioglu A, Arnold F (2013). Measuring coverage in MNCH: tracking progress in health for women and children using DHS and MICS household surveys. PLoS Med.

[20] Whiting K (2019). 5 facts to know about Africa's powerhouse - Nigeria.

[21] United Nations Office for the Coordination of Humanitarian Affairs (2018). North-East Nigeria: Humanitarian Situation Update, December 2017.

[22] Momoh Z (2018). Development partners, humanitarian assistance and quest for reconstruction of North-Eastern Nigeria. Int J Soc Sci and Hum Rev.

[23] Leidman E, Tromble E, Yermina A (2017). Acute malnutrition among children, mortality, and humanitarian interventions in conflict-affected regions – Nigeria, October 2016-March 2017. Morb and Mort Weekly Reports.

[24] Edwards J (2017). The challenge of humanitarian response in conflict: Nigeria Case Study.

[25] United Nations Office for the Coordination of Humanitarian Affiars (2018). Northeast Nigeria health sector response strategy, 2017-2018.

[26] Newbarander W, Ickx P, Feroz F, Stanekzai H (2014). Afghanistan’s basic package of health services: its development and effects on rebuilding the health system. Global Public Health.

[27] World Health Organization (2020). Early warning, alert and response system (EWARS).

[28] World Health Organization (2020). EWARS- improving early detection and prompt response to acute public health events.

[29] World Health Organization (2020). Health Resources Availability Monitoring System (HeRAMS).

[30] Bawa S, Shuaib F, Saidu M, Ningi A, Abdullahi S, Abba B, et al. Conduct of vaccination in hard-to-reach areas to address potential polio reservoir areas, 2014-2015. BMC Public Health. 2018;18. 10.1186/s12889-018-6194-y PMC6291919 30541501

[31] Strachan CE, Kana M, Martin S, Dada J, Wandera N, Marasciulo M (2016). The use of formative research to inform the design of a seasonal malaria chemoprevention intervention in northern Nigeria. Malaria J.

[32] World Health Organization (2020). Adamawa launches first ever Malaria chemoprevention campaign.

[33] World Health Organization. Northeast Nigeria response (Borno State). Health Sector Bull. 2017;24.

[34] Denue BA, Akawu CB, Kwayabura SA, Kida I (2018). Low case-fatality during 2017 cholera outbreak in Borno State, North Eastern Nigeria. Ann Afr Med.

[35] United Nations Office for the Coordination of Humanitarian Affiars (2020). Nigeria: measles outbreak - Oct 2016.

[36] World Food Programme (2018). National Conversation on the Humanitarian Development: Peace NexusBeyond the Immediate North East Humanitarian Crisis.

