# Correction to: The relationship between armed conflict and reproductive, maternal, newborn and child health and nutrition status and services in northeastern Nigeria: a mixed-methods case study

**DOI:** 10.1186/s13031-021-00348-7

**Published:** 2021-03-01

**Authors:** Jennifer A. Tyndall, Khadidiatou Ndiaye, Chinwenwo Weli, Eskedar Dejene, Nwanneamaka Ume, Victory Inyang, Christiana Okere, John Sandberg, Ronald J. Waldman

**Affiliations:** 1 American University of Nigeria, Yola, Nigeria; 2 Milken Institute School of Public Health, George Washington University, Washington, DC, USA; 3 Doctors of the World – USA, New York, NY USA

**Correction to: Confl Health 14, 75 (2020)**

**https://doi.org/10.1186/s13031-020-00318-5**

Following publication of the original article [1], the authors identified an error in the author name of Chinwenwo Weli

- The incorrect author name is: Chinwewo Weli
- The correct author name is: Chinwenwo Weli

The author group has been updated above and the original article [1] has been corrected.
